# M2BPgs-HCC: An Automated Multilectin Bead Array Indicating Aberrant Glycosylation Signatures Toward Hepatitis C Virus-Associated Hepatocellular Carcinoma Prognosis

**DOI:** 10.3390/molecules29235640

**Published:** 2024-11-28

**Authors:** Hiroko Shimazaki, Haruki Uojima, Kazumi Yamasaki, Tomomi Obayashi, Sayaka Fuseya, Takashi Sato, Masashi Mizokami, Atsushi Kuno

**Affiliations:** 1 Molecular & Cellular Glycoproteomics Research Group, Cellular and Molecular Biotechnology Research Institute, National Institute of Advanced Industrial Science & Technology, Tsukuba 305-8565, Japan; hiroko.shimazaki@pss.co.jp (H.S.); t-obayashi@aist.go.jp (T.O.); fuseya-sa@aist.go.jp (S.F.); takashi-sato@aist.go.jp (T.S.); 2 Precision System Science Co., Ltd., Matsudo 271-0064, Japan; 3 Genome Medical Science Project, National Center for Global Health and Medicine, Ichikawa 272-8516, Japan; lb-uojima@hospk.ncgm.go.jp (H.U.); mmizokami@hospk.ncgm.go.jp (M.M.); 4 Department of Gastroenterology, Internal Medicine, Kitasato University School of Medicine, Sagamihara 252-0375, Japan; 5 Clinical Research Center, NHO National Hospital Organization Nagasaki Medical Center, Omura 856-0835, Japan; rsr03768@nifty.com

**Keywords:** M2BPGi, hepatocellular carcinoma, glycosylation, lectin array

## Abstract

Regular monitoring of patients with a history of hepatitis C virus (HCV) infection is critical for the detection and management of hepatocellular carcinoma (HCC). Mac-2 binding protein glycosylation isomer (M2BPGi) has been used to monitor fibrosis progression and predict HCC. However, HCC prediction based on M2BPGi has not been optimized. Here, we identified HCC risk-related glycan signatures of M2BP using a newly developed automated bead array with multiplexed lectins. Among 955 patients with HCV who achieved sustained virological response following direct-acting antiviral treatment, we compared M2BP glycosylation from sera of 42 patients diagnosed with HCC during follow-up and 43 without HCC (control) by the lectin microarray. At the HCC observation point, we found significant differences in 17 lectins. Using an automated bead array with 12 of 17 lectins, a principal component analysis (PCA) biplot differentiated HCC from control, along the PC1 axis, explaining 75.2% of variance. Based on PC1, we generated a scoring formula for an HCC-related glycosylation signature on M2BP (M2BPgs-HCC), showing good diagnostic performance for HCC (*p* = 2.92 × 10^−8^, AUC = 0.829). This automated multilectin bead array improved the ability of M2BP to detect HCC, providing a candidate test for HCC surveillance in combination with other HCC markers.

## 1. Introduction

Liver cancer is the sixth most frequent cancer worldwide with approximately 870,000 new cases in 2022 (https://gco.iarc.fr/, accessed on 1 July 2024). Hepatocellular carcinoma (HCC) accounts for the highest rate of liver cancer cases and one of the major risk factors for HCC development is hepatitis C virus (HCV) infection [1,2]. Chronic inflammation and liver damage caused by HCV infection can lead to the development of cancerous cells in the liver over time. Early detection and treatment of HCV infection can reduce the risk of developing HCC [1,2]. Direct-acting antivirals (DAAs) have markedly improved the management of chronic HCV infections [3].

Ongoing monitoring is essential in patients with a history of HCV infection, particularly those with advanced liver fibrosis or cirrhosis. Even after successful HCV treatment, these patients have a high risk of developing HCC, and it is crucial to promptly identify and treat HCC to improve the prognosis [4]. Regular surveillance using imaging studies, such as ultrasound, computed tomography (CT), or magnetic resonance imaging (MRI), along with blood tests can help identify signs of HCC at an early stage. Alpha-fetoprotein (AFP), AFP-L3%, protein induced by vitamin K absence-II (PIVKA-II), and other serum biomarkers have been used; however, they are inadequate in terms of sensitivity or specificity, especially for early HCC detection [5,6]. Recently, various combinations of diagnostic methods have been evaluated, such as GAAD and GALAD [7,8]. The GALAD score, which combines gender and age with three biomarkers (AFP-L3, AFP, and Des-gamma carboxyprothrombin or PIVKA-II), has progressed to phase 3 biomarker validation [9].

Mac-2 binding protein glycosylation isomer (M2BPGi), which is M2BP with disease-relevant glycosylation recognized by the plant lectin *Wisteria floribunda* (WFA), has emerged as a novel biomarker for liver fibrosis in clinical practice [10,11]. M2BP is a highly glycosylated protein with an advantage in the easy detection of glycan alteration [10]. Its ability to assess or predict liver fibrosis is well-established in different chronic liver diseases or after virus treatment [12,13,14]. Moreover, there is growing evidence that elevated M2BPGi levels are associated with an increased risk of developing HCC [15,16,17,18]. Sasaki et al. showed that in patients who achieved sustained virological response (SVR) following interferon treatment, the median M2BPGi value of the HCC group was significantly higher than that of the non-HCC group [19]. Harimoto et al. conducted a study that revealed that M2BPGi predicts the recurrence of HCC after hepatectomy in patients with HCC who achieved SVR [20]. Generally, glycan structures depend on producing cells [21], and there is a potential relationship between M2BP glycan and cancer, as determined by cell-based assays [22,23,24]. However, little is known about the relationship between the detailed M2BP glycan structure and cancer. Therefore, we investigated aberrant glycosylation of M2BP associated with HCC (M2BPgs-HCC), providing candidate biomarkers for disease surveillance.

## 2. Results

### 2.1. Patient Flow and Baseline Characteristics

**Figure 1:**
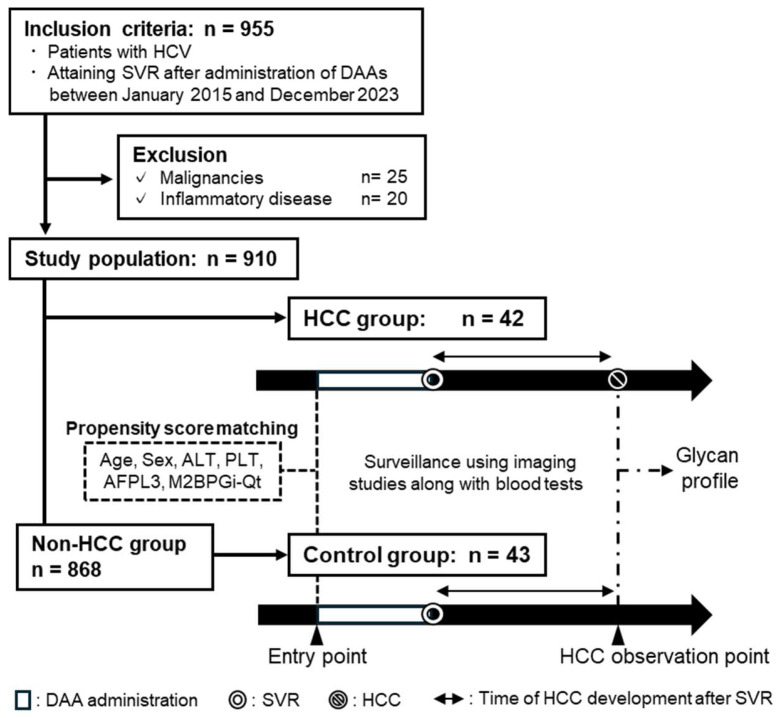
Study flow chart.

In total, 910 patients with HCV who achieved SVR following DAA treatment were observed over a median period of 1542 days. During this period, 42 patients were diagnosed with HCC and assigned to the HCC group. By conducting propensity score matching at the entry point, 43 patients who did not develop HCC were included in the control group (Figure 1). There were no significant differences in age, sex, alanine transaminase (ALT), AFP, M2BPGi quantity (M2BPGi-Qt) levels, or liver cirrhosis between the two groups at the entry point (Table 1).

### 2.2. Clinical Characteristics of the HCC Group at the HCC Observation Point

The clinical characteristics of the two groups at the HCC observation point (for the HCC group) or its equivalent (for the control group) are shown in Supplementary Table S1 and in Table 2. The mean M2BPGi-Qt levels in the HCC group were significantly higher than those of the control group (M2BPGi-Qt HCC group: 3.24 ± 3.1, control group: 1.44 ± 0.7, *p* = 0.001). Albumin levels, AFP-L3%, and PIVKA-II also showed significant differences between the HCC and control groups (*p* = 1.89 × 10^−4^, *p* = 6.26 × 10^−5^, and *p* = 0.021, respectively). AFP-L3% counts for 23 patients with HCC were zero (Supplementary Table S1).

**Figure 2:**
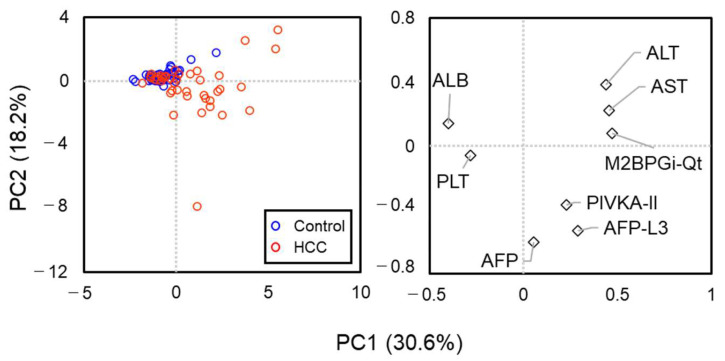
Principal component analysis of clinical characteristics at the HCC observation point. Blue and red circles represent controls and patients with HCC, respectively. The right graph shows the eigenvectors of eight parameters.

Figure 2 shows the results of a principal component analysis (PCA) based on eight parameters (AST, ALT, PLT, ALB, AFP, AFP-L3%, PIVKA-II, and M2BPGi-Qt). The PCA could not clearly distinguish between the HCC and control groups; the control group and a portion of patients with HCC showed a relatively homogeneous distribution, while other patients with HCC were spread in the direction explained by ALT/AST /M2BPGi-Qt or AFP/AFP-L3/PIVKA-II. These results suggest that there is a group of patients with HCC that cannot be adequately characterized by existing HCC markers, highlighting the importance of incorporating M2BPGi into the scoring system for HCC detection. The similarity between the two groups was also evaluated using PERMANOVA, which showed slightly significant differences between the HCC and control groups: *p*-value < 0.0001, but the pseudo-F value is low (pseudo-F = 1.43).

### 2.3. HCC-Related Glycan Feature Extraction of M2BP in Serum Samples from Patients

Next, we performed glycan feature profiling of M2BP to characterize aberrant glycosylation on M2BP associated with HCC. The M2BPs were immunoprecipitated from 1 μL of sera of enrolled patients and subjected to the 45-plexed lectin microarray analysis. The lectins and their binding specificities are shown in Supplementary Table S2. We first confirmed that the mean of 45 lectin signals per sample well correlated with signal intensities in Western blotting of the same eluate. The obtained lectin signals are summarized in Table 3. Significant differences (*p* < 0.001) were found in 17 lectin signals between the HCC and control groups (Table 3 and Supplementary Figure S1).

Briefly, asialoglycan-binding lectins, such as ECA (12), *Bauhinia purpurea* lectin (BPL, 23), TJA-II (24), and WFA (33), were chosen as the preferred binders for the M2BPs of the HCC group. In contrast, α2,6-sialylation-binding lectins, such as SNA (8) and TJA-I (10), were chosen as the preferred binders for the control group. Notably, WFA had a similar score (*p* = 2.14 × 10^−4^ in Table 3) to that of M2BPGi-Qt (*p* = 6.79 × 10^−4^), indicating the reliability of the multiplexed system. Additional glycosylation signatures of fucose-binding lectins [*Pisum sativum* agglutinin (PSA, 2), *Lens culinaris* agglutinin (LCA, 3), *Aspergillus oryzae* lectin (AOL, 5), and *Aleuria aurantia* lectin (AAL, 6)] in the HCC group and the α2,3-sialylation-binding lectin ACG (21) in the control group were obtained, suggesting the advantage of multiple lectins.

### 2.4. Automatic Glycofeature Detection of Serum M2BP Using a 12-Plexed Lectin Bead Array

**Figure 3:**
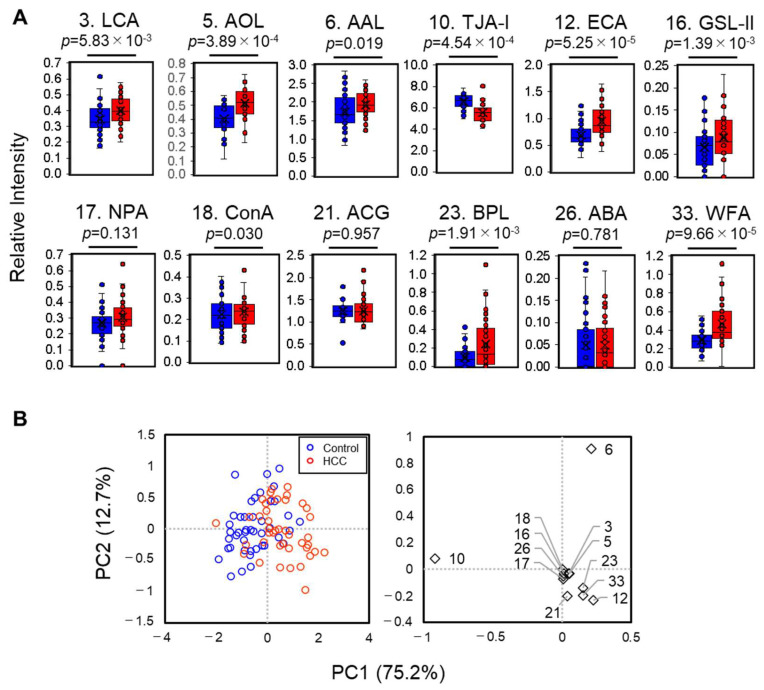
Automatic glycofeature detection of serum M2BP using a 12-plexed lectin bead array. (**A**) Univariate analysis of lectin signals in control (blue) and HCC (red) groups. (**B**) Principal component analysis using 12-lectin signals for serum M2BP. The right graph shows eigenvectors of 12 lectins, where each lectin is indicated by an ID number listed in Table 3.

In addition to the 45-plexed lectin microarray analysis, we also adopted a fully automated method involving a multiplexed lectin bead array, GlycoBIST, as described in our previous report [27]. Among the 17 lectins, 3 with overlapping glycan specificity (PSA (2) for LCA (3), TJA-II (24) for BPL (23), and SNA (8) for TJA-I (10)) and 2 with higher noise in GlycoBIST (*Datura stramonium* agglutinin (DSA, 15) and *Urtica dioica* agglutinin (UDA, 29)) were excluded from the final design. The bead array format used in this study comprised 12-lectin beads (Table 3), with positive/negative beads as described by Fuseya et al. [28], as well as an anti-M2BP antibody bead as a target indicator. The reaction time was optimized by measuring 85 HCV-SVR samples under three conditions (5, 10, and 30 min). Supplementary Figure S2 shows typical peak charts, indicating an optimal reaction time of 10 min. The box-and-whisker plot for each lectin signal was consistent with that of the lectin microarray using the 16-h reaction (Figure 3A). The PCA biplot using these 12 lectin signals well separated the HCC and control groups, especially along the PC1 axis, which explained 75.2% of the total variance (Figure 3B). PERMANOVA also showed highly significant differences (*p*-value < 0.0001, pseudo-F = 25.8) between the HCC and control groups, indicating that combined lectin information was better than existing markers (pseudo-F = 1.43). Therefore, based on PC1, we generated a scoring formula for an HCC-related glycosylation signature on M2BP (M2BPgs-HCC).

### 2.5. Statistical Characterization of the 12-Plexed Lectin Bead Array

**Figure 4:**
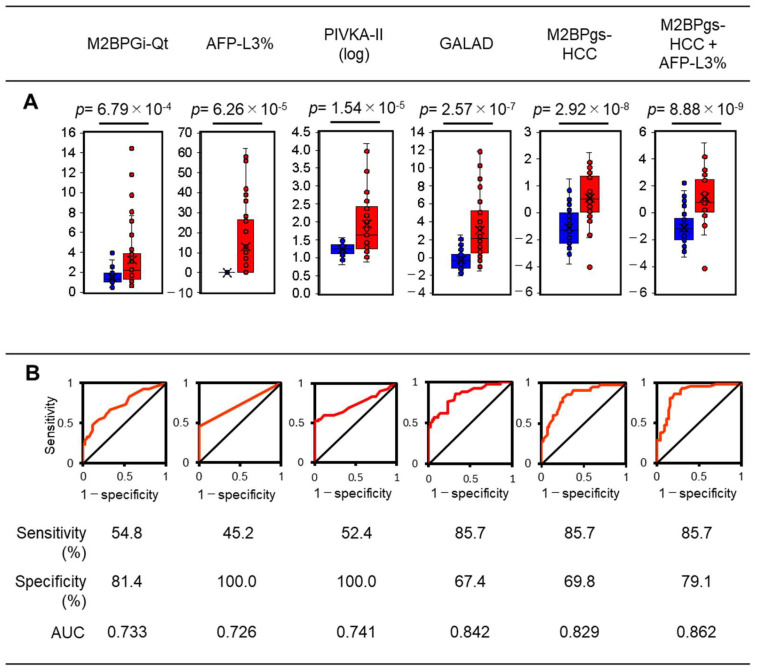
Comparison of the predictive value of M2BPGi-Qt, AFP-L3%, PIVKA-II, GALAD, M2BPgs-HCC, and the combination of M2BPgs-HCC and AFP-L3%. (**A**) Box-and-whisker diagram. Blue and red circles indicate individual patient values. (**B**) ROC curves are shown by red line.

Figure 4 shows the results of univariate analyses with Student’s *t*-tests and ROC curves, indicating a good HCC diagnostic score for M2BPgs-HCC [*p* = 2.92 × 10^−8^, AUC = 0.829, sensitivity = 85.7%, specificity = 69.8%]. The M2BPgs-HCC score was superior to that of M2BPGi-Qt [*p* = 6.79 × 10^−4^, AUC = 0.733, sensitivity = 54.8%, specificity = 81.4%], confirming that the use of multiplexed lectin and WFA drastically improves the predictive value of M2BP for HCC. The results were further compared with those for approved HCC markers and a recently proposed combined score, the GALAD score, which has recently advanced to phase 3 validation [9]. The M2BPgs-HCC score showed much better performance than that of AFP-L3% [*p* = 6.26 × 10^−5^, AUC = 0.726, sensitivity = 45.2%, specificity = 100%] or log10[PIVKA-II] [*p* = 1.54 × 10^−5^, AUC = 0.741, sensitivity = 52.4%, specificity = 100%]. The GALAD score also showed good performance [*p* = 2.57 × 10^−7^, AUC = 0.842, sensitivity = 85.7%, specificity = 67.4%]; however, it is worth noting that the results were comparable to those for M2BPgs-HCC, derived from a single molecule. Based on the PCA plot (Figure 2) and ROC curves (Figure 4), M2BPgs-HCC had different characteristics from those of AFP-L3%, suggesting that the combination of M2BPgs-HCC with AFP-L3% may improve the diagnostic score. As we expected, the M2BPgs-HCC + AFP-L3% score, obtained as PC1 in the PCA plot, effectively detected HCC with *p* = 8.88 × 10^−9^, AUC = 0.862, sensitivity = 85.7%, and specificity = 79.1% (Figure 4).

## 3. Discussion

This study showed that multiplexed lectin detection to fine-tune the glycosylation signature of M2BP (M2BPgs) improved the value of M2BP for HCC surveillance in patients with HCV-SVR. Two different multilectin assays revealed an M2BPgs-HCC featuring fucose and asialoglycan. Fucosylation is one of the most important types of glycosylation occurring during carcinogenesis [29,30]. Fucosylation of M2BP is also associated with cancer [24]. Alternatively, the asialo-*N*-glycan of M2BP is a feature of M2BPGi established as a liver fibrosis glycobiomarker, which bears a highly branched form with WFA-binding ability, whereas fully α2,6-sialylated M2BP is predominant in healthy volunteers [31]. Interestingly, asialo-form M2BP, which is expressed in stromal cells, such as hepatic stellate cells [32], is associated with increased aggressiveness of HCC by activating mTOR signaling with galectin-3 lattice formation [23]. ST6GAL1 overexpression in HCC cells suppresses metastasis by inhibiting the galectin-3 interaction with cell surface MCAM, whose glycosylation is converted from asialo-form to sialo-form [22]. Using established HCC cell lines [33,34], we recognized the expression of M2BP in HCC cell (Supplementary Figure S3), and we propose that not only fibrogenic cells but also HCC cells may express asialo-M2BP, potentially contributing to carcinogenesis and cancer proliferation. Further studies are needed to identify the cellular origin of circulating asialo-M2BP.

The discovery of omics-based glycan biomarkers has been driven by mass spectrometry, liquid chromatography, and capillary electrophoresis. For clinical applications, that is, the detection of these glycan biomarkers, multilectin-based technology has contributed significantly [35]. However, lot-to-lot variation and manual operations have hindered the use of conventional technologies, such as lectin microarrays, in clinical applications. GlycoBIST was a cutting-edge analytical chemistry tool for glycobiology, and the recently developed milli-bead-based lectin array can be used for automatic lectin-dependent glycan characterization [27]. The present study represents the first clinical application of GlycoBIST. Unlike most previous glycobiomarker assays, this method can be used to evaluate biomarkers from the verification phase using multiplexed lectins. This approach facilitates efficient glycobiomarker development, identifying new glycosylation signatures. Further improvements in terms of robustness are needed. As shown in Figure 4, there was one discrepant count indicating an undesirable reduction in some lectin signals due to measurement failure. We obtained an appropriate count in the re-measurement, resulting in more precise differentiation, as shown in Supplementary Figure S4.

This study had some limitations. First, the sample size was not large enough to fully evaluate the performance of M2BPgs-HCC, especially for ROC analyses. Although more than 900 patients were observed, the final number of patients in test groups was limited to fewer than 100 owing to the study design. In addition, our results were derived from a single study population. Further validation of M2BPgs-HCC on a large scale including other populations is required to evaluate the robustness of the present model or to design a more effective scoring formula for M2BPgs-HCC.

## 4. Materials and Methods

### 4.1. Patients and Study Design

#### 4.1.1. Study Population

A multicenter study was conducted at three locations in Japan. The inclusion criteria for this study were patients with HCV who achieved sustained virological response (SVR) between January 2015 and December 2023, defined as the absence of detectable HCV RNA for an extended period following treatment. Regular follow-up assessments were performed, including evaluation of tumor biomarkers (PIVKA-II and AFP) and imaging scans at intervals of 6–12 months. Patients with a history of HCC or HCC detectable on ultrasonography at entry, severe inflammatory diseases, or malignancies unrelated to the study focus were excluded. Liver cirrhosis, a type of liver fibrosis, was diagnosed through lab results and imaging tests showing an irregular liver surface, collateral portosystemic channels, ascites, or esophageal varices. A liver biopsy was not performed due to associated risks and inaccuracies.

#### 4.1.2. Study Design

The HCC group consisted of patients who developed HCC during the follow-up period. The control group consisted of patients who did not develop HCC during follow-up. The patients in the control group were matched to patients with HCC using propensity score matching based on age, sex, ALT, PLT, AFP-L3%, and M2BP-Gi quantity (M2BPGi-Qt) at the entry point. AFP and AFP-L3 levels were measured using a μTAS Wako i30 fully automated immunoanalyzer (Fujifilm Wako Pure Chemical Corporation, Osaka, Japan). AFP-L3% was calculated as the ratio of AFP-L3 to AFP. When AFP-L3 was under the lower limit of detection, AFP-L3% was defined as 0%. The M2BPGi-Qt levels were measured using a fully automated HISCL-5000 Immunoassay Kit (Sysmex Co., Kobe, Japan) as described previously [36]. PIVKA-II levels were determined using an Architect immunoassay system (Abbot Japan LLC, Tokyo, Japan) based on the principle of chemiluminescent immunoassays.

### 4.2. Immunoprecipitation of M2BP

An anti-human M2BP polyclonal antibody (R&D Systems, Inc., Minneapolis, MN, USA) was purified with Protein G using MonoSpin ProG (GL Sciences Inc., Tokyo, Japan) and biotinylated using a Biotin Labeling Kit-NH2 (Dojindo Laboratories, Kumamoto, Japan). Then, 1 μL of HCV-SVR serum was mixed with 100 ng of biotinylated anti-M2BP antibody in a total of 45 μL of 1% Triton X-100 in Tris-buffered saline (TBSTx) and shaken for 30 min at 4 °C. Then, 100 μg of pre-washed magnetic beads conjugated with streptavidin was added, and the mixture was shaken for another 30 min. After three washes of the beads with TBSTx, the bound molecules were eluted in 0.1 M glycine-HCl with 1% Triton X-100 (pH 2.7) for 5 min with shaking and neutralized by adding a one-tenth volume of 1 M Tris-HCl (pH 9.0). PBS was used as an immunoprecipitation-negative control (IP-NC).

### 4.3. Differential Glycan Analysis of M2BP

An antibody-overlay lectin microarray was performed, as described previously with slight modifications [37]. Briefly, one-fifth of immunoprecipitation eluate, equivalent to 0.2 μL serum which is considered to contain approximately 3–5 ng of M2BP based on the average value for healthy subjects, was diluted to 60 μL with PBS containing 1% Triton X-100 (PBSTx) and then applied to the lectin microarray (Precision System Science Co., Ltd., Matsudo, Japan). After overnight incubation at 20 °C, 20 μg of human serum polyclonal IgG was added to the chip and incubated for 30 min. After washing three times with PBSTx, 100 ng of biotinylated anti-M2BP antibody in 60 μL of PBSTx was added and incubated for 60 min. After three rounds of washing with PBSTx, 200 ng of Cy3-labeled streptavidin (GE Healthcare Technologies Inc., Chicago, IL, USA) in 60 μL of PBSTx was added and incubated for 30 min. After washing three times with PBSTx, the glass slides were scanned using an evanescent-field fluorescence scanner (Glycostation Reader 2300; GlycoTechnica Ltd., Yokohama, Japan). The scan data were digitized and quantified using GlycoStation ToolsPro version 1.5 (GlycoTechnica Ltd.), and the average signal intensity of the three spots after subtracting the background intensity was used in the following process as the net signal intensity. Each lectin signal was expressed as a normalized value with a mean of 45 net lectin signals.

### 4.4. Multilectin Bead Array Analysis

A multilectin bead array was performed using a LuBEA-VIII device (Precision System Science Co., Ltd.). A GlycoBIST tip was designed using 12 selected lectin beads, anti-M2BP antibody beads, and biotinylated BSA (positive) and BSA (negative) beads. The GlycoBIST tip was fabricated as described previously [27]. Briefly, the probe beads were constructed by immobilization with appropriate proteins, followed by drying. Spacer beads were prepared by washing with 0.1% Triton X-100 in PBS and drying. They were then packed together in a dedicated capillary tip (Precision System Science Co., Ltd.) according to the design. For the multilectin bead array analysis, half of the immunoprecipitated M2BP, equivalent to 0.5 μL serum that was considered to contain approximately 10 ng of M2BP based on the average value for healthy subjects, was diluted to 160 μL with TBSTx and combined with 40 μL of probing buffer (1% Triton X-100, 0.5 M glycine, 1 mM CaCl_2_, 1 mM MnCl_2_ in TBS). The biotinylated anti-M2BP antibody was diluted to 0.5 μg/mL with CanGet Signal Solution I (Toyobo Co., Ltd., Osaka, Japan), and HRP-conjugated streptavidin (Proteintech Group, Inc., Rosemont, IL, USA) was diluted to 0.5 μg/mL with CanGet Signal Solution II (Toyobo Co., Ltd.). Subsequently, the prepared GlycoBIST tips, M2BP samples, primary and secondary antibody solutions, mixed substrate solutions (Bio-Rad Laboratories, Inc., Hercules, CA, USA), and wash buffer (0.1% Triton X-100 in TBS) were placed into the LuBEA-VIII device for automatic measurement using a predefined program [27]. The obtained lectin signals were subtracted from both the BSA signal and the appropriate lectin signal of the IP-NC sample. The obtained “net” lectin signals are expressed as normalized values for an average of 12 lectins.

### 4.5. Statistical Analysis

SPSS (version 24.0; IBM Corp., Armonk, NY, USA) and Excel version 2404 were used for data analyses. The baseline characteristics of each dataset are presented as medians, with interquartile ranges for continuous variables. Categorical variables are presented as percentages. The Student’s *t*-test or Welch’s test was used to compare baseline characteristics or lectin signals between datasets. The distribution of categorical variables was compared using Fisher’s exact or chi-square tests, as appropriate. A value of *p* < 0.05 was considered statistically significant. A principal component analysis (PCA) was performed using R version 4.3.2. For the data from the lectin microarray or multilectin bead array analysis, the mean normalized values were used without scaling among lectin signals. For the comparisons of clinical parameters, the data were scaled in advance. To evaluate the similarity between the HCC and control groups, we used PERMANOVA implemented in scikit-bio 0.6.2. In PERMANOVA, distance matrices were constructed using Euclidean distances, and a permutation test with 9999 iterations was performed.

## Figures and Tables

**Table 1:** Baseline characteristics of patients with the HCC and control groups at the entry point.

Clinical Characteristics		HCC	Control	*p*-Value
Number of patients		42	43	
Age	Years	69.9 ± 9.8	69.1 ± 9.9	0.713
Gender: Male	*n* (%)	21 (50.0)	23 (53.5)	0.829
Liver cirrhosis	*n* (%)	26 (61.9)	25 (58.1)	0.826
DAA ^a^: DCA ^b^ + ASV ^c^/SOF ^d^ + RBV ^e^/SOF + LDV ^f^/OBV ^g^ + PTV ^h^-r ^i^/EG ^j^/GP ^k^	*n*	9/1/28/0/0/4	4/3/31/1/2/2	0.241
DM	*n* (%)	7 (16.7)	9 (20.9)	0.787
Alcohol	*n* (%)	6 (14.3)	6 (14.0)	0.782
Aspartate aminotransferase (AST)	IU/L	59.0 ± 31.9	54.5 ± 24.6	0.474
Alanine aminotransferase (ALT)	IU/L	47.5 ± 28.1	55.1 ± 27.0	0.204
Platelet (PLT)	10^3^/μL	12.0 ± 5.7	12.8 ± 5.7	0.506
Serum albumin (ALB)	g/dL	3.7 ± 0.5	3.8 ± 0.3	0.404
AFP	ng/mL	17.2 ± 27.1	11.5 ± 15.3	0.239
AFP-L3%	%	1.5 ± 2.7	1.0 ± 1.9	0.374
M2BPGi-Qt	AU/mL	5.75 ± 4.1	5.80 ± 4.5	0.964

^a^ DAA; Direct-acting antivirals, ^b^ DCA; Daclatasvir, ^c^ ASV; Asunaprevir, ^d^ SOF; Sofosbuvir, ^e^ RBV; Ribavirin, ^f^ LDV; Ledipasvir, ^g^ OBV; Ombitasvir, ^h^ PTV; Paritaprevir, ^i^ r; Ritonavir, ^j^ EG; Elbasvir + Grazoprevir, ^k^ GP; Glecaprevir + Pibrentasvir.

**Table 2:** Baseline characteristics at the HCC observation point (HCC) or its equivalent (control).

Clinical Characteristics		HCC	Control	*p*-Value
Number of patients		42	43	
Time of HCC development after SVR	Days	1202 ± 697	1272 ± 635	0.803
AST	IU/L	35.7 ± 24.2	27.4 ± 21.6	0.117
ALT	IU/L	25.0 ± 24.3	19.1 ± 10.7	0.150
PLT	10^3^/μL	13.5 ± 5.7	15.6 ± 5.1	0.082
ALB	g/dL	4.0 ± 0.5	4.3 ± 0.3	<0.001
AFP	ng/mL	1896 ± 11.8	2.5 ± 2.1	0.304
AFP-L3%	%	12.9 ± 18.7	0	<0.001
M2BPGi-Qt	AU/mL	3.24 ± 3.1	1.44 ± 0,7	0.001
PIVKA-II	mAU/mL	1196 ± 3280	18 ± 6.8	0.021

**Table 3:** The result of lectin microarray of immunoprecipitated M2BP.

ID	Lectin	Rough Lectin Binding ^a^	Relative Intensity ^b^			For GlycoBIST ^d^
			Control (95% CI)	HCC (95% CI)	*p*-Value ^c^	
1	LTL	fucose	0.00 (0.00–0.00)	0.00 (0.00–0.00)	1.22 × 10^−2^	
2	PSA	fucose	0.21 (0.20–0.23)	0.27 (0.25–0.29)	1.71 × 10^−4^	
3	LCA	fucose	0.37 (0.35–0.40)	0.47 (0.43–0.50)	6.32 × 10^−5^	⭘
4	UEA-I	fucose	0.02 (0.02–0.03)	0.03 (0.02–0.04)	1.26 × 10^−1^	
5	AOL	fucose	0.22 (0.20–0.24)	0.38 (0.34–0.42)	6.34 × 10^−10^	⭘
6	AAL	fucose	1.43 (1.33–1.53)	1.82 (1.71–1.94)	1.74 × 10^−6^	⭘
7	MAL-I	sialic acid	0.71 (0.65–0.78)	0.78 (0.71–0.85)	1.70 × 10^−1^	
8	SNA	sialic acid	3.37 (3.26–3.47)	3.04 (2.88–3.20)	8.30 × 10^−4^	
9	SSA	sialic acid	3.39 (3.29–3.49)	3.21 (3.03–3.39)	7.62 × 10^−2^	
10	TJA-I	sialic acid	4.22 (4.08–4.36)	3.70 (3.51–3.90)	5.41 × 10^−5^	⭘
11	PHA(L)	complex N-glycan	0.70 (0.64–0.77)	0.85 (0.76–0.95)	9.94 × 10^−3^	
12	ECA	LacNAc	0.39 (0.36–0.42)	0.55 (0.48–0.62)	2.32 × 10^−4^	⭘
13	RCA120	LacNAc	4.19 (4.12–4.27)	4.03 (3.94–4.12)	7.34 × 10^−3^	
14	PHA(E)	complex N-glycan	3.60 (3.44–3.77)	3.46 (3.31–3.61)	1.95 × 10^−1^	
15	DSA	complex N-glycan	5.65 (5.48–5.81)	5.06 (4.92–5.20)	8.39 × 10^−7^	
16	GSL-II	GlcNAc	0.05 (0.04–0.05)	0.06 (0.05–0.06)	6.55 × 10^−4^	⭘
17	NPA	mannose	0.08 (0.07–0.08)	0.09 (0.08–0.10)	7.09 × 10^−4^	⭘
18	ConA	mannose	0.14 (0.13–0.16)	0.31 (0.27–0.35)	2.14 × 10^−10^	⭘
19	GNA	mannose	0.02 (0.02–0.03)	0.02 (0.02–0.02)	9.54 × 10^−1^	
20	HHL	mannose	0.03 (0.02–0.03)	0.04 (0.03–0.04)	1.11 × 10^−2^	
21	ACG	Gal	3.24 (3.11–3.37)	2.82 (2.69–2.95)	1.22 × 10^−5^	⭘
22	TxLC-I	complex N-glycan	2.54 (2.39–2.69)	2.54 (2.40–2.68)	9.90 × 10^−1^	
23	BPL	Gal	0.16 (0.14–0.19)	0.36 (0.26–0.46)	4.27 × 10^−4^	⭘
24	TJA-II	fucose	0.19 (0.17–0.20)	0.25 (0.22–0.29)	4.29 × 10^−4^	
25	EEL	Gal	0.23 (0.12–0.34)	0.20 (0.09–0.31)	6.80 × 10^−1^	
26	ABA	complex N-glycan	0.20 (0.17–0.22)	0.12 (0.11–0.13)	3.43 × 10^−8^	⭘
27	LEL	LacNAc	4.65 (4.44–4.85)	4.50 (4.28–4.73)	3.48 × 10^−1^	
28	STL	LacNAc	2.59 (2.46–2.71)	2.70 (2.58–2.83)	1.72 × 10^−1^	
29	UDA	GlcNAc	0.42 (0.38–0.45)	0.53 (0.50–0.57)	1.02 × 10^−5^	
30	PWM	GlcNAc	0.00 (0.00–0.01)	0.01 (0.00–0.01)	3.96 × 10^−2^	
31	Jacalin	O-glycan	0.01 (0.01–0.02)	0.01 (0.01–0.01)	5.34 × 10^−1^	
32	PNA	O-glycan	0.00 (0.00–0.00)	0.00 (0.00–0.00)	ND	
33	WFA	GalNAc	0.43 (0.37–0.49)	0.83 (0.64–1.02)	2.14 × 10^−4^	⭘
34	ACA	O-glycan	0.00 (0.00–0.00)	0.00 (0.00–0.00)	2.82 × 10^−1^	
35	MPA	O-glycan	0.00 (0.00–0.01)	0.00 (0.00–0.01)	2.90 × 10^−1^	
36	HPA	O-glycan	0.05 (0.02–0.07)	0.11 (0.04–0.19)	1.04 × 10^−1^	
37	VVA	GalNAc	0.00 (0.00–0.00)	0.00 (0.00–0.00)	4.27 × 10^−2^	
38	DBA	GalNAc	0.00 (0.00–0.00)	0.00 (0.00–0.00)	3.38 × 10^−1^	
39	SBA	GalNAc	0.00 (0.00–0.00)	0.00 (0.00–0.00)	ND	
40	Calsepa	Mannose	0.51 (0.46–0.55)	0.62 (0.56–0.68)	3.89 × 10^−3^	
41	PTL-I	fucose	0.00 (0.00–0.00)	0.00 (0.00–0.00)	ND	
42	MAH	sialic acid	0.00 (0.00–0.00)	0.00 (0.00–0.00)	ND	
43	WGA	GlcNAc	0.99 (0.86–1.12)	1.21 (1.11–1.31)	8.58 × 10^−3^	
44	GSL-IA_4_	Gal	0.00 (0.00–0.00)	0.00 (0.00–0.00)	ND	
45	GSL-IB_4_	Gal	0.00 (0.00–0.00)	0.00 (0.00–0.00)	ND	

^a^ Detailed binding specificities are shown in Supplementary Table S2, both based on Bojar D et al., 2022 [25], Shilova N et al., 2024 [26], and Lectin frontier database. ^b^ Values are shown as the relative intensity to the average of 45 lectin (1.0 = average of 45 lectin signals) and represented in two decimal places. ^c^ Red indicates a significant difference (*p* < 0.001) between the control and HCC groups. ^d^ The circle indicates those used in automatic lectin bead array.

## Data Availability

The raw data can be obtained on request from the corresponding author.

## Supplementary Materials

The following supporting information can be downloaded at: https://www.mdpi.com/article/10.3390/molecules29235640/s1, Table S1: Clinical characteristics at the HCC observation point (HCC) or its equivalent (control); Table S2: List of lectins used in this study and their binding specificity.; Figure S1: Univariate analysis of lectin signals in the lectin microarray for serum M2BP.; Figure S2: Optimization of the reaction time for GlycoBIST measurement of serum-derived M2BP.; Figure S3: Expression of M2BP and AFP in HCC cell lines.; Figure S4: Statistical characterization of M2BPgs-HCC and the combination of M2BPgs-HCC and AFP-L3% after re-measuring one sample with a discrepant count.
