# Supplementary material for: M2BPgs-HCC: An Automated Multilectin Bead Array Indicating Aberrant Glycosylation Signatures Toward Hepatitis C Virus-Associated Hepatocellular Carcinoma Prognosis
Source: Molecules. 2024 Nov 28;29(23):5640. doi: 10.3390/molecules29235640 (PMC11643838; doi:10.3390/molecules29235640)
Supplement: Supplementary file 1 [file molecules-29-05640-s001.zip › molecules-3297046-supplementary.pdf]

Supplementary Table S1. Clinical characteristics at the HCC observation point (HCC) or its equivalent (control).

Control

No	AST	ALT	PLT	ALB	AFP	AFPL3%	M2BPGi	PIVKA-II
1	18	17	19.4	4.3	2.9	0.0	0.79	28.6
2	28	19	20.0	4.9	2.0	0.0	0.48	17.3
3	21	21	12.4	4.5	1.7	0.0	1.14	25.0
4	24	15	9.3	4.1	1.8	0.0	2.69	10.0
5	13	12	18.9	4.4	1.6	0.0	1.05	15.7
6	23	25	5.4	4.1	0.8	0.0	0.45	36.2
7	35	21	16.6	4.3	5.6	0.0	1.50	10.4
8	16	9	7.4	4.4	1.4	0.0	1.23	16.9
9	46	27	12.4	4.2	10.2	0.0	1.34	12.4
10	26	19	11.5	4.3	1.6	0.0	1.36	24.0
11	46	36	17.8	4.5	1.3	0.0	1.10	6.4
12	19	8	12.7	4.4	1.9	0.0	2.55	17.2
13	22	27	12.4	4.3	2.8	0.0	1.01	11.9
14	29	15	18.0	3.5	1.8	0.0	2.02	13.2
15	22	15	14.7	4.8	0.7	0.0	0.53	24.0
16	33	27	9.7	4.4	11.3	0.0	3.25	11.6
17	149	45	25.0	3.9	0.7	0.0	1.31	29.7
18	56	54	9.2	4.4	3.8	0.0	0.77	16.6
19	23	16	13.8	4.9	1.3	0.0	0.68	36.9
20	16	16	18.7	4.5	1.8	0.0	1.01	18.1
21	19	13	17.6	4.7	2.5	0.0	2.03	18.6
22	16	7	16.5	3.6	1.4	0.0	1.04	22.9
23	18	13	12.3	4.7	1.0	0.0	3.91	22.3
24	28	30	11.5	4.2	3.6	0.0	1.30	19.3
25	25	15	17.5	4.0	3.8	0.0	1.34	16.1
26	21	16	9.6	4.8	2.0	0.0	1.37	11.6
27	23	15	18.0	4.0	1.9	0.0	1.12	27.6
28	20	15	14.0	4.5	1.9	0.0	1.34	8.9
29	20	11	18.0	4.1	1.7	0.0	1.86	10.7
30	16	15	29.6	4.8	3.8	0.0	0.60	20.6
31	16	18	21.0	4.1	1.3	0.0	0.80	25.6
32	49	39	17.8	4.6	5.0	0.0	0.65	20.6
33	16	11	15.3	4.2	1.2	0.0	1.44	24.2
34	42	32	16.3	4.5	2.5	0.0	1.08	22.1
35	4	9	21.0	4.8	0.8	0.0	0.88	21.8
36	21	17	25.5	4.2	1.1	0.0	1.02	20.1
37	25	17	11.9	4.8	2.6	0.0	2.19	13.6
38	18	12	15.0	4.1	1.4	0.0	1.44	14.1
39	21	9	17.1	4.5	2.0	0.0	1.93	16.9
40	16	11	18.4	4.3	1.6	0.0	2.13	18.4
41	27	21	5.9	4.1	3.2	0.0	1.69	11.2
42	23	12	11.9	4.0	3.8	0.0	1.94	17.4
43	27	18	21.5	4.3	1.8	0.0	2.80	21.8

HCC

No	AST	ALT	PLT	ALB	AFP	AFPL3%	M2BPGi	PIVKA-II
1	21	16	18.6	4.6	4.0	0.0	1.09	19.7
2	39	12	17.5	4.4	76,520	58.2	1.18	3441.9
3	40	26	5.2	3.9	2.1	0.0	4.06	10.9
4	31	17	10.3	4.2	166.0	35.9	2.35	13.1
5	27	21	10.0	3.1	3.3	0.0	4.30	231.3
6	33	20	10.0	3.6	21.7	37.3	3.78	99.9
7	115	41	21.0	3.9	657.4	37.3	3.50	12466.0
8	25	17	15.0	4.2	9.4	7.0	3.09	775.5
9	33	19	9.9	4.2	112.6	55.9	2.61	2615.7
10	73	34	9.7	3.1	289.1	10.3	2.28	7.8
11	19	9	15.0	3.8	4.7	0.0	1.80	3237.6
12	4	5	17.0	4.6	2.7	0.0	1.26	26.6
13	21	9	23.0	3.5	2.4	13.0	1.95	156.0
14	57	59	11.0	4.6	7.2	62.1	2.45	18.8
15	31	23	4.0	4.2	6.8	0.0	1.08	422.3
16	23	21	13.5	4.2	2.6	0.0	1.31	43.9
17	26	16	12.9	4.0	5.8	0.0	1.67	77.0
18	29	23	8.3	3.5	2.2	25.9	8.07	149.5
19	23	18	18.0	4.7	0.9	0.0	0.82	63.3
20	112	110	7.6	3.9	4.8	0.0	9.74	28.7
21	28	13	15.4	4.5	2.8	0.0	1.05	21.9
22	27	14	6.8	4.3	13.3	28.1	3.18	17.6
23	27	15	8.7	4.2	2.9	0.0	7.12	14.9
24	30	18	8.4	3.4	1.5	0.0	4.15	14979.9
25	21	26	5.1	3.0	55.6	22.1	11.79	48.8
26	29	21	16.8	4.5	1.8	0.0	1.26	236.3
27	24	15	13.7	4.2	1.6	0.0	1.12	73.7
28	26	15	23.4	4.2	1.1	0.0	2.58	688.7
29	24	16	9.8	5.0	3.2	0.0	0.47	17.5
30	20	11	15.2	4.5	2.3	0.0	1.39	10.5
31	23	21	15.4	4.2	11.3	0.0	1.47	12.6
32	35	16	18.0	4.3	2.5	0.0	1.16	21.7
33	30	26	22.8	3.9	1314.3	20.6	2.09	19.6
34	17	11	14.6	4.3	0.9	0.0	1.31	13.0
35	22	14	2.8	3.1	31.8	1.5	1.76	9531.9
36	24	14	14.1	3.6	14.0	38.9	7.70	42.8
37	29	15	12.0	3.6	73.2	29.0	5.76	117.6
38	84	125	10.9	4.1	211.3	13.6	2.05	21.0
39	27	18	17.1	3.7	3.3	0.0	2.70	17.7
40	54	33	27.1	4.1	2.3	0.0	2.41	11.8
41	11	4	21.8	3.4	34.6	41.9	0.64	379.6
42	87	70	8.6	3.5	38.3	3.8	14.45	32.5

Supplementary Table S2. List of lectins used in this study and their binding specificity.

ID	Lectin	LMA[a]	GB[b]	Binding specificity[c]
		17	12	
1	LTL			Fucα1-3(Galβ1-4)GlcNAc (Lewis x), Fucα1-2Galβ1-4GlcNAc (H-type 2)
2	PSA	○		Fucα1-6GlcNAc (Core Fuc) , High-Man
3	LCA	○	○	(GlcNAcβ1-2Manα1-)$_2$-3,6-Manβ1-4GlcNAc$_2$β, Fucα1-6GlcNAc (Core Fuc), High-Man
4	UEA_I			Fucα1-2Galβ1-4GlcNAc (H-type 2)
5	AOL	○	○	Fucα1-6GlcNAc (Core Fuc), Fucα1-2Galβ1-4GlcNAc (H-type 2)
6	AAL	○	○	Fucα1-3(Galβ1-4)GlcNAc (Lewis x), Fucα1-6GlcNAc (Core Fuc)
7	MAL_I			Siaα2-3Galβ1-4GlcNAc
8	SNA	○		Siaα2-6Gal/GalNAc
9	SSA			Siaα2-6Gal/GalNAc
10	TJA-I	○	○	Siaα2-6Gal/GalNAc
11	PHA(L)			tri/tetra-antennary complex-type *N*-glycan
12	ECA	○	○	Galβ1-4GlcNAc
13	RCA120			Galβ1-4GlcNAc
14	PHA(E)			*N*-glycans with outer Gal and bisecting GlcNAc
15	DSA	○		(GlcNAcβ1-4)n (Chitin), tri/tetra-antennary *N*-glycan
16	GSL-II	○	○	agalactosylated tri/tetra antennary glycans, GlcNAc
17	NPA	○	○	High-Mannose including Manα1-6Man
18	ConA	○	○	High-Mannose including Manα1-6(Manα1-3)Man
19	GNA			High-Mannose including Manα1-3Man
20	HHL			High-Mannose including Manα1-3Man or Manα1-6Man
21	ACG	○	○	Siaα2-3Galβ1-4GlcNAc
22	TxLC_I			Man$_3$ core, bi/tri-antennary *N*-glycans, GalNAc
23	BPL	○	○	Galβ1-3GalNAc, GalNAc
24	TJA-II	○		Fucα1-2Galβ1, GalNAcβ1
25	EEL			Gala1-3Galβ1-4GlcNAc, Fucα1-2(Galβ1-3)GlcNAc (H antigen)
26	ABA	○	○	Galβ1-3GalNAc, GlcNAc
27	LEL			(GlcNAcβ1-4)n (Chitin), (Galβ1-4GlcNAc)n (polylactosamine)
28	STL			(GlcNAcβ1-4)n (Chitin) (GlcNAcβ1-4MurNAc)n (peptidoglycan backbone)
29	UDA	○		GlcNAcβ1-4GlcNAc (Chitin), Man5 ~ Man9
30	PWM			(GlcNAcβ1-4)n (Chitin)
31	Jacalin			Galβ1-3GalNAc, αGalNAc (6*O*-unsubstituted)
32	PNA			Galβ1-3GalNAc
33	WFA	○	○	GalNAcβ1-4GlcNAc, Galβ1-3(-6)GalNAc
34	ACA			Galβ1-3GalNAc
35	MPA			αGalNAc, Galβ1-3GalNAc
36	HPA			αGalNAc
37	VVA			αGalNAc, GalNAcα1-3Gal
38	DBA			GalNAcα1-3GalNAc (Blood group A), GalNAcα1-3GalNAc
39	SBA			GalNAc, GalNAcα1-3Gal, GM1 ganglioside
40	Calsepa			High-Mannose (Man$_{2-6}$), *N*-glycans including bisecting GlcNAc
41	PTL_I			αGalNAc
42	MAH			Siaα2-3Galβ1-3(Siaα2-6)GalNAc, sulfated glycan
43	WGA			(GlcNAcβ1-4)n (Chitin), NeuAc
44	GSL_I_A4			αGalNAc
45	GSL_I_B4			αGal

[a]17 out of 45 lectins showing significant differences between HCC and control groups in the lectin microarray analysis are marked. [b]12 out of 17 lectins used in GlycoBIST are circled.
[c]The information is based on Bojar D et at, 2022 [25], Shilova N et al, 2024 [26], and Lectin frontier database (LfDB - Lectin Frontier DataBase)

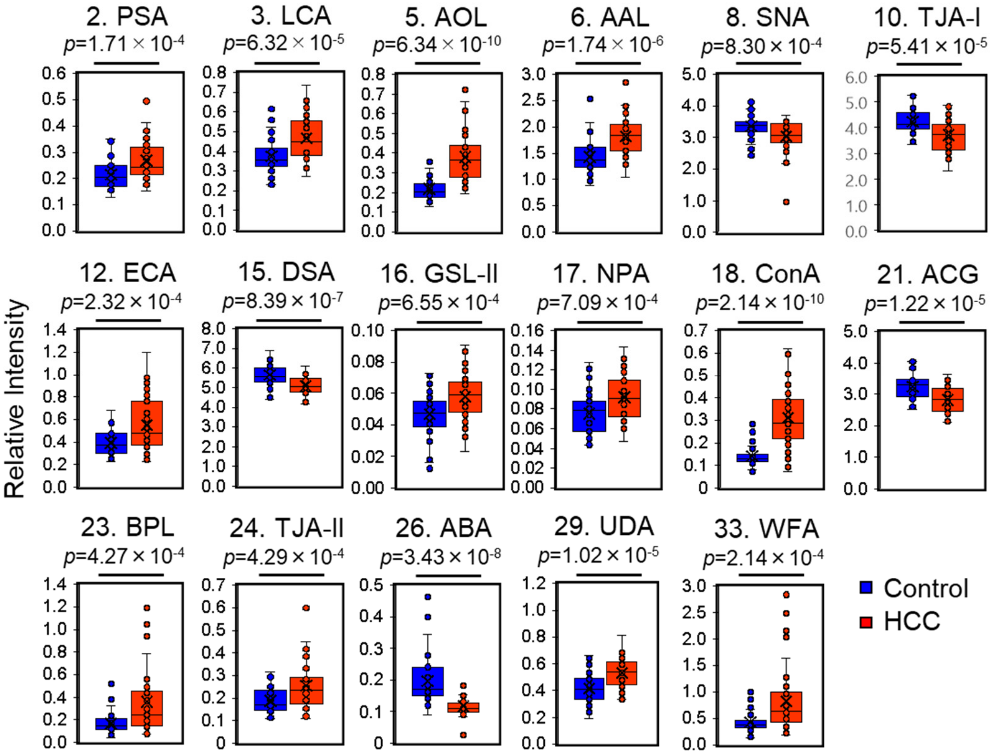

Supplementary Figure S1. Univariate analysis of lectin signals in the lectin microarray for serum M2BP.

The result of 17 out of 45 lectins, which show significant differences (p < 0.001) between the control and HCC groups, are presented.

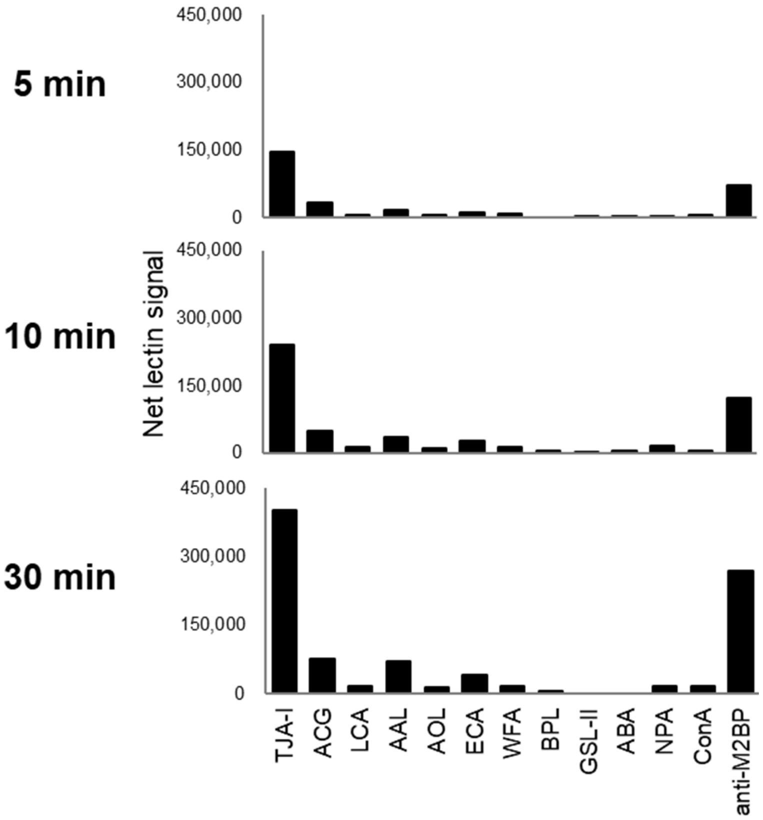

Supplementary Figure S2. Optimization of the reaction time for GlycoBIST measurement of serum derived M2BP.

The representative results of GlycoBIST measurement with reaction time of 5, 10, 30 min are shown.

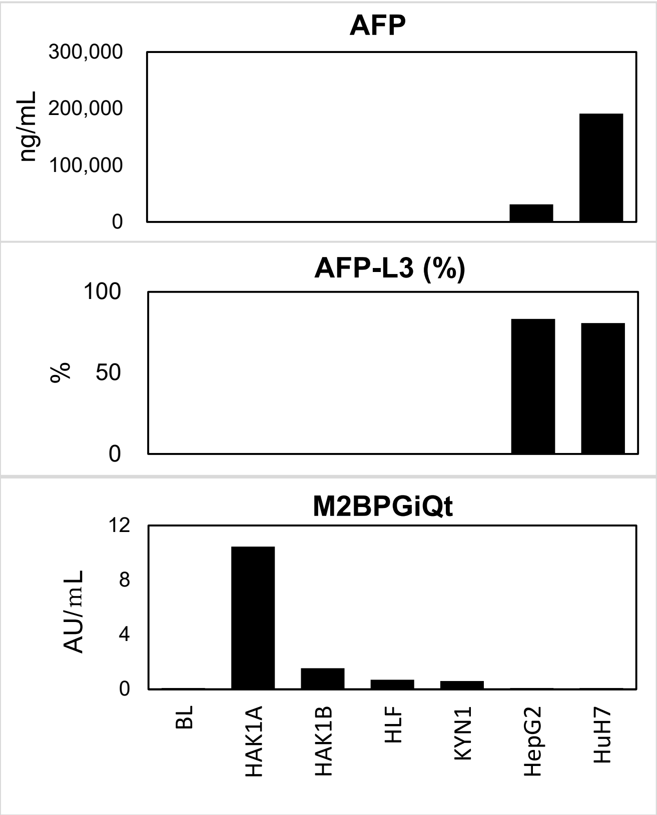

Supplementary Figure S3. Expression of M2BP and AFP in HCC cell lines.

Levels of AFP, AFP-L3%, and M2BPGi in the culture supernatant are shown. BL: serum-free DMEM.

The HCC cell lines HepG2, Huh7, and HLF, obtained from Riken or JCRB Cell Bank, were cultured in Dulbecco's modified Eagle's medium (DMEM; Thermo Fisher Scientific Inc.) or RPMI1640 medium (Thermo Fisher Scientific Inc.) supplemented with 10% heat-inactivated fetal bovine serum (FBS) and antibiotics and maintained at 37 °C in an incubator with 5% CO_2. The HAK1A, HAK1B, and KYN-1 HCC cell lines were cultured in RPMI1640 medium with 10% heat-inactivated FBS and antibiotics. After reaching 60−80% confluence, the cells were washed three times with Dulbecco's phosphate-buffered saline (D-PBS) and further cultured in FBS- and antibiotic-free DMEM or RPMI1640 medium for 48 h. After incubation, the culture supernatants were collected and filtered through a 0.45-μm disc filter. M2BPGi-Qt, AFP, and AFP-L3% were measured following the same methods used for serum samples.

	M2BPgs-HCC	M2BPgs-HCC + AFP-L3%

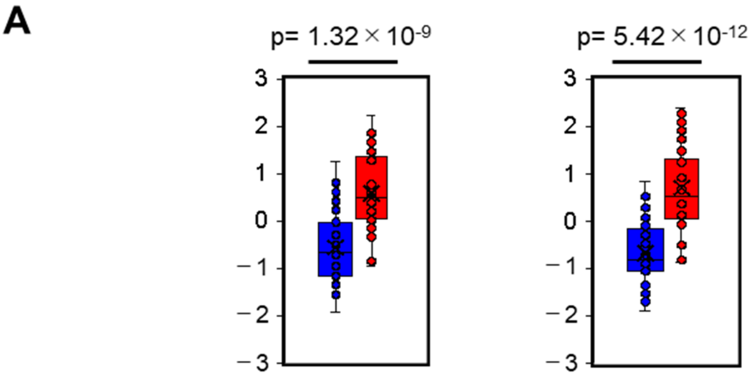

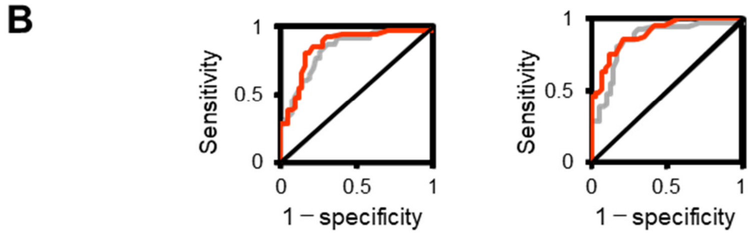

	M2BPgs-HCC	M2BPgs-HCC + AFP-L3%
Sensitivity (%)	87.8	85.4
Specificity (%)	72.1	81.4
AUC	0.850	0.896

Supplementary Figure S4. Statistical characterization of M2BPgs-HCC and the combination of M2BPgs-HCC and AFP-L3% after re-measuring a sample with a discrepant count.

(A) Univariate analyses. Blue and red indicates control and HCC group, respectively. (B) ROC analyses. Gray lines indicate the original data (same as Figure 4) and red lines indicate the data after re-measuring.
